# Knowledge and Attitudes Regarding Antibiotic Use Among Adult Consumers, Adult Hispanic Consumers, and Health Care Providers — United States, 2012–2013

**DOI:** 10.15585/mmwr.mm6428a5

**Published:** 2015-07-24

**Authors:** Louise K. Francois Watkins, Guillermo V. Sanchez, Alison P. Albert, Rebecca M. Roberts, Lauri A. Hicks

**Affiliations:** 1 Epidemic Intelligence Service, CDC; 2 Division of Bacterial Diseases, National Center for Immunization and Respiratory Diseases, CDC

Appropriate antibiotic use, in particular avoidance of antibiotics for upper respiratory infections likely to be caused by viruses (1,2), is a key component of efforts to slow the increase in antibiotic-resistant infections (3). Studies suggest that Hispanic consumers might differ from non-Hispanic consumers in their knowledge and attitudes regarding antibiotic use (4). To better understand health care provider and consumer knowledge and attitudes that influence antibiotic use, CDC analyzed national internet survey data collected from participants living in the United States during 2012–2013. The participants represented three groups: 1) the total population of adult consumers (all ethnicities); 2) adult Hispanic consumers; and 3) health care providers. Hispanic consumers were more likely than all consumers to believe that if they have a cold, antibiotics would help them to get better more quickly (48% versus 25%), and more likely to obtain antibiotics not prescribed by a clinician, such as antibiotics left over from a previous illness (25% versus 9%), obtained from a neighborhood grocery store (23% versus 5%), or obtained from a friend or family member (17% versus 6%). Most providers surveyed (54%) reported that they believed their patients expect antibiotics during visits for a cough or cold, whereas 26% of all consumers reported this expectation. To maximize knowledge about appropriate antibiotic use among outpatients in the United States, public health initiatives should target Hispanic as well as general audiences.

Porter Novelli, a public relations firm, administers national surveys (conducted online since 2011) to health care providers and adult consumers living in the United States to assess health-related knowledge and attitudes. Questions pertaining to antibiotic use in the setting of cough or cold were included in the Summer 2012 HealthStyles and Fall 2013 HealthStyles surveys (all consumers), the 2013 Estilos survey (Hispanic consumers), and the 2012 DocStyles survey (providers).

HealthStyles survey participants were recruited from market research firm GfK’s Knowledge Panel ( http://www.gfk.com/us/Solutions/consumer-panels/Pages/GfK-KnowledgePanel.aspx ), which uses both random-digit–dialed and address-based sampling methods to reach potential participants (laptop computer and Internet access are provided to participants if needed). Estilos survey participants were recruited via the QueOpinas ( http://www.offerwise.com ) research panel, which uses television advertisements in both English and Spanish to recruit panel members. Estilos survey questions were written in English and translated into Spanish; both language versions were available to Estilos participants. HealthStyles responses were weighted using U.S. Current Population Survey data ( http://www.bls.gov/cps/ ) and Estilos responses were weighted using U.S. Census American Community Survey data ( http://www.census.gov/programs-surveys/acs/ ) to match U.S. proportions according to sex, age, household income, household size, education, census region. HealthStyles responses were also weighted by race/ethnicity, metropolitan statistical area, and internet access; Estilos responses were also weighted by country of origin and acculturation status (based on years living in the United States, language spoken at home, cultural self-identification, and use of Spanish language media). DocStyles survey participants were recruited from the Epocrates Honors and Epocrates Allied Health panels. Surveyed providers were primary care physicians and nurse practitioners. Eligibility criteria for providers included an active individual, group, or hospital-based practice in the United States, and at least 3 years of work experience. Physician participants mirrored the American Medical Association master file proportions for age, sex, and region. Survey respondents were reimbursed for time and effort with nominal cash or cash-equivalent awards.

Surveys were distributed to 4,703 U.S. consumers in 2012 (response rate = 86%; n = 4,044), 4,420 U.S. consumers in 2013 (response rate = 79%; n = 3502), 2,609 Hispanic consumers (response rate = 38%; n = 1,000), and 3,149 health care providers (response rate = 48%, n = 1,503, including 1,001 adult and family physicians, 252 nurse practitioners, and 250 pediatricians). Responses to questions asked of all consumers and Hispanic consumers were compared.

Responses among Hispanic consumers differed markedly from those among all consumers (Table 1). Hispanic consumers were more likely to agree that when they have a cold, antibiotics prevent more serious illness (40% versus 17%) and help them get better more quickly (48% versus 25%). Hispanic consumers were also more likely to report obtaining antibiotics from sources other than their doctor or clinic, including leftover antibiotics from a prior illness (25% versus 9%); from a neighborhood grocery store (23% versus 5%); or from a family member or friend (17% versus 6%). Although knowledge of antibiotic side effect profiles was generally comparable between Hispanic consumers and all consumers, Hispanic consumers were less aware of potential dangers of antibiotic use, such as antibiotics becoming less effective after their use (antibiotic resistance) or that antibiotics might kill the “good” bacteria the body needs.

Fifty-four percent of providers perceived that patients expect an antibiotic at a visit for a viral illness (Table 2); 26% of all consumers and 41% of Hispanic consumers reported expecting an antibiotic at a visit for a cough or cold (Table 1). However, despite the substantial percentage of consumers in both groups reporting the expectation of an antibiotic, all consumers most commonly expected reassurance (42%) and Hispanic consumers most commonly expected suggestions for symptom relief (58%). Providers were most commonly deterred from prescribing antibiotics because of the potential for antibiotic resistance (94%) and side effects or allergic reactions (71%), and most commonly considered experience with the drug (85%), cost (84%), and side effect profile (81%) when deciding which antibiotic to prescribe (Table 2).

## Discussion

Health care providers face ongoing challenges in responding to patient expectations regarding antibiotic use. Antibiotics are rarely indicated for a cough or cold (1,2), but 41% of Hispanic consumers and more than a quarter of all consumers reported expecting an antibiotic at a health care visit for these syndromes. More than half of providers reported that their patients expect an antibiotic prescription. Provider perception of patient expectations for an antibiotic is important, because it has been shown to be a reliable predictor of overprescribing (5–7), which might contribute to preventable side effects, adverse drug events and antibiotic resistance. However, the fact that consumers were more likely to expect reassurance (all consumers) or suggestions for symptom relief (Hispanic consumers) suggests that provider counseling, rather than an antibiotic prescription, is paramount in consumer satisfaction.

Knowledge about the effectiveness of antibiotics in the setting of a cough or cold was limited among both consumer populations, but particularly among Hispanic consumers. Hispanic consumers were almost three times more likely than all consumers to report obtaining antibiotics from a source other than a clinic or pharmacy with a prescription from a health care provider. These results are consistent with findings from previous regional studies, which reported that, compared with non-Hispanic whites, Hispanic consumers were less knowledgeable about indications for antibiotic use, were more frequently dissatisfied if antibiotics were not prescribed (8), and commonly obtain antibiotics without a prescription (4,9). Despite federal laws mandating prescriptions, antibiotics might be readily available over the counter in some locally owned grocery stores serving Hispanic communities (6,10). Self-administration of antibiotics is concerning, as it might result in a lack of appropriate care for those who avoid seeing a provider, and might also lead to increased antibiotic resistance in areas with over-the-counter antibiotic availability.

The findings in this report are subject to at least five limitations. First, despite efforts to reach consumers from all social strata, potential participants without a phone line, stable address, or sufficient literacy were likely excluded, resulting in a biased sample. Second, low survey response rates, particularly among Hispanic consumers and providers, might further have contributed to selection bias. Third, unweighted data demonstrated that participants in the all-consumer sample were disproportionately white/non-Hispanic and educated compared with the general population; Hispanic consumer respondents were younger, more educated, and had lower household income than the national Hispanic population; and provider respondents were disproportionately male and from the Northeast and West regions of the United States. Fourth, the sample of all consumers included Hispanic respondents (accounting for 13.3% and 14.1% of the weighted responses in the 2012 HealthStyles and 2013 HealthStyles surveys, respectively), which might underestimate the magnitude of differences between Hispanic and non-Hispanic groups. Finally, important differences might exist between Hispanic subgroups (based on acculturation status or ethnic origin) that were not captured in this study.

Appropriate antibiotic use is important to limit unnecessary adverse drug events and development of antibiotic resistance. The differences in health knowledge and attitudes between Hispanic and all consumers observed in this study underscore the importance of considering cultural factors in public health messaging about appropriate antibiotic use. Research is needed to investigate the influence of specific cultural factors, such as immigration status, country of origin, and degree of acculturation, on health knowledge and attitudes among Hispanic as well as other minority populations. Public health initiatives have traditionally focused on the patient-provider relationship as a framework to disseminate educational messages and interventions. However, these approaches might miss consumers who use antibiotics not prescribed for them. Complementary interventions that improve access to health care, particularly among recent immigrants who might face the steepest barriers to receiving health care, might also help reduce antibiotic self-administration (4).

Patient expectations for antibiotics and provider perceptions of these expectations highlight the ongoing need for consumer education and improvement of patient-provider communication to maximize judicious antibiotic prescribing. CDC’s “Get Smart: Know When Antibiotics Work” program ( http://www.cdc.gov/GetSmart/Community ) distributes culturally appropriate materials in both English and Spanish to assist providers in communicating with patients about when and why antibiotics are indicated.

**Summary**

What is already known on this topic?

Antibiotic resistance is a growing public health concern. Appropriate antibiotic use is a key strategy to address increases in antibiotic-resistant infections. Consumers’ and providers’ knowledge and attitudes toward antibiotic use influence their expectations and prescribing behaviors.

What is added by this report?

Hispanic consumers in the United States are almost twice as likely as consumers overall to believe that taking antibiotics lessens the symptoms of a cold, and almost three times as likely to obtain antibiotics not prescribed by a clinician, such as antibiotics left over from a previous illness. Fifty-four percent of surveyed health care providers think their patients expect antibiotics during visits for a cough or cold; 26% of surveyed consumers report having this expectation.

What are the implications for public health practice?

Consumer education needs to emphasize both the limited circumstances in which respiratory infections require an antibiotic and the individual and population-level harms of inappropriate antibiotic use. Public health initiatives might target Hispanic as well as general audiences and consider cultural differences in health knowledge and attitudes, for example, through the use of culturally appropriate materials made available in both English and Spanish.

## Figures and Tables

**TABLE 1:** Knowledge and attitudes about antibiotic use among Hispanic consumers compared with all consumers — United States, 2012–2013*

Question/Responses	Hispanic consumers† (%)	All consumers§ (%)	Percentage point difference¶
When I have a cold, I should take antibiotics to prevent getting a more serious illness.**	(40)††	(17)††	(23)
When I have a cold, antibiotics help me to get better more quickly.**	(48)††	(25)††	(23)
**Which of the following side effects are common after taking an antibiotic?** §§			
Nausea/Vomiting	(22)	(22)	(0)
Diarrhea	(21)	(35)	(14)
Abdominal or stomach pain	(19)	(22)	(3)
Headache	(15)	(10)	(5)
Rash	(12)	(14)	(2)
None of these	(27)	(16)	(11)
Don’t know	(25)	(40)	(15)
**If you get antibiotics from sources other than your doctor or clinic, where do you get them?** §§			
Leftover from being sick before	(25)	(9)	(16)
Neighborhood grocery store	(23)	(5)	(18)
Family member or friend	(17)	(6)	(11)
From another country	(8)	(2)	(6)
Internet pharmacy	(7)	(1)	(6)
Some other source not listed	(7)	(3)	(4)
I have never done this	(46)	(80)	(34)
**What do you expect of your provider during a visit for cough or cold?** **			
Suggestions for symptom relief	(58)	(35)	(23)
An antibiotic	(41)	(26)	(15)
Rule out something worse/Reassurance	(31)	(42)	(11)
Other	(2)	(3)	(1)
None of these	(5)	(16)	(11)
Not sure	(6)	(15)	(9)

* Based on 2013 Estilos survey (Hispanic consumers) and Summer 2012 HealthStyles and Fall 2013 HealthStyles surveys (all consumers). Individual consumer response numbers are not reported because percentages have been weighted to be nationally representative.

† n = 1,000.

§ n = 4,044 (Summer 2012 HealthStyles survey) and n = 3,502 (Fall 2013 HealthStyles survey).

¶ Because of the large sample sizes, p-values are not reported.

** Data on all consumers are from the Summer 2012 HealthStyles survey.

†† Percentages reflect those who reported they agreed with the statement.

§§ Data on all consumers are from the Fall 2013 HealthStyles survey.

**TABLE 2:** Knowledge and attitudes about antibiotics and antibiotic resistance among health care providers — United States, 2012*

Question/Responses	Health care providers† (%)
**What do parents/patients expect of you during a visit for a viral illness?**	
Symptom relief recommendation/OTCs	(77)
Rule out secondary infection/reassurance	(72)
An antibiotic	(54)
None of these	(<1)
Not sure	(2)
**Which potential risks deter you from prescribing antibiotics?**	
Antibiotics become less effective (antibiotic resistance)	(94)
Side effects and allergic reactions	(71)
May kill “good” bacteria that your body needs	(58)
None of these	(2)
Not sure	(<1)
**When you prescribe an antibiotic, which of the following do you consider when deciding which antibiotic to prescribe?**	
Experience prescribing that drug	(85)
Cost	(84)
Side effect profile	(81)
Guidelines/Literature	(74)
Convenient dosing	(73)
Patient request	(23)
Pharmaceutical sales representative information	(6)
Other	(4)
None of these	(0)
Do not prescribe antibiotics	(1)

**Abbreviation:** OTCs = over-the-counters (i.e., drugs available without a prescription).

* Based on 2012 DocStyles survey (health care providers).

† n = 1,503.
